# Epidemiologic and Health Economic Evaluation of Cervical Cancer Screening in Rural China

**DOI:** 10.31557/APJCP.2020.21.5.1317

**Published:** 2020-05

**Authors:** Fei Zhao, Ying Wen, Yang Li, Siyuan Tao, Li Ma, Yuqian Zhao, Le Dang, Ying Wang, Fanghui Zhao, Jinghe Lang, Youlin Qiao, Chun-xia Yang

**Affiliations:** 1 *West China School of Public Health and West Fourth Hospital, Sichuan University, Chengdu, China. *; 2 *Department of Molecular Epidemiology, Shenzhen Center for Disease Control and Prevention, Shenzhen,China. *; 3 *National Cancer Center/National Clinical Research Center for Cancer/Cancer Hospital, Chinese Academy of Medical Sciences and Peking Union Medical College, Beijing, China. *; 4 *Institute of Medical Information, Chinese Academy of Medical Sciences and Peking Union M Medical College, Beijing, China. *; 5 *Dalian Medical University, Dalian, China. *; 6 *Sichuan Cancer Hospital &Institute, Sichuan Cancer Center, School of Medicine, University of Electronic Science &Technology of China, Chengdu, China. *; 7 *Peking Union Medical college Hospital, Chinese Academy of Medical Sciences/Peking Union Medical College(CAMS/PUMC), Beijing, China. *

**Keywords:** Cervical cancer, screening, rural China

## Abstract

**Background:**

Cervical cancer is preventable and curable by detected early and managed effectively. To explore the most economical and effective cervical cancer screening strategies would lay a solid foundation for reducing the health and economic burden of cervical cancer.

**Methods:**

A Markov model was established for a cohort of 100,000 female to simulate the natural history of cervical cancer. 18 screening strategies were estimated including careHPV, Thin prep cytologic (TCT), Visual inspection with acetic acid/ Lugol’s iodine (VIA / VILI), careHPV in series with VIA / VILI, careHPV in series with TCT, three methods parallel connection every 1, 3, 5 years respectively. Model outcomes included cumulative risk of incidence and death of cervical cancer, quality-adjusted life years (QALYs), cost-effectiveness ratios (CERs), incremental cost-effectiveness ratios (ICERs), cost-utility ratios (CURs) and benefits.

**Results:**

According to the results of epidemiological analysis, careHPV similar to the parallel connection every 1 year achieved highest epidemiological effects via reducing the cumulative risk of onset and death by more than 98 %. In health-economic terms, CER among all the screening strategies ranged from -756.34 to 113040.3 Yuan per year and CUR ranged from -169.91 to 11968.27 Yuan per QALY. The benefit ranged from -1629 to 996 Yuan. The incremental cost-effectiveness analysis showed that three methods in parallel every 1 year, TCT every 1 year, VIA/VILI every 1, 3, 5 years and careHPV every 5 years were dominant strategies.

**Conclusion:**

Considering the economic and health benefits of all the strategies, our results suggested careHPV every 3 or 5 years and VIA/VILI every 1 or 3 years eventually were more appropriate as screening methods in rural China.

## Introduction

Cervical cancer is the fourth most common cancer among women globally, with 570,000 new cases and 311,000 deaths in 2018 (Ferlay et al., 2018). Age standardized incidence and mortality rates was much higher in underdeveloped countries across the world (13.1, 6.9 per 100,000 respectively), given the high cost of establishing screening programs and current low screening coverage. About 84% of the new cases and 85% of the deaths occurred in low and middle-income countries (Ferlay et al., 2018). China accounts for approximately one fifth of the world population, and its cervical cancer burden has a substantial effect on global estimate of the current and future burden of the disease. A national survey showed about 99,000 new cases and 31,000 death of cervical cancer in China 2015, approximately accounting for 18% and 11% that of the world respectively (Chen et al., 2016). 

Cervical cancer is one of the most preventable and curable forms of cancer, as long as it is detected early and managed effectively. Thus, it is of great significance to carry out early screening, early diagnosis and early treatment to make considerable reduction of cervical events. Various screening programs have been established across the world in recent decades and have already made great accomplishment. Developed countries launched national cervical cancer screening at the end of the 20^th^ century. According to statistics, the incidence of cervical cancer has been reduced by 70% through nationwide screening, and the mortality rate of cervical cancer among women aged 20-40 has been reduced by more than 80% (Becker et al., 2003). However, developing countries, involving China, have not established a standardized and comprehensive cervical cancer screening system for target population. Disease burden remains high, considering the large population, insufficient coverage and poor quality of screening test, especially in rural (Di et al., 2015; Olson et al., 2016). In recent years, our country has been working on a national screening program, named “two cancers screening”. This project launched in rural areas from 2009 to 2011 completed the cervical cancer screening of 11.69 million women in three years. Since 2012, the screening scope of the program has been expanded to 10 million rural women per year (Qiao et al., 2015). Traditional pap smears and VIA/VILI were mainly used in this program given to the low cost. However, poor accuracy and high requirements of medical technology lead to the traditional one was replaced by TCT gradually. Since the HPV virus is the leading cause of cervical cancer, corresponding detection methods are mounting, including careHPV, which went public in China 2012. Given it’s accuracy, convenience, economics, it has been gradually applied in lagging areas. Owing to merits and demerits in various methods, evaluation which methods will yield the greatest epidemiological and economic effectiveness in our target population is truly critical. But studies in this filed were still lacking, we only found two related researches in this filed conducted in rural Shandong (Zong et al., 2015) and some poverty-stricken regions in China respectively (Xie et al., 2017). For the former one, a total of 3,763 women were enrolled and screened by pap smear,VIA/VILI and careHPV respectively. The result supported careHPV alone was a more appropriate method for rural areas concerning higher specificity and sensitivity. A total of 3,086 women volunteered in the later one. They conducted a health economic analysis comparing three methods:VIA/VILI, TCT, and HPV test and suggested VIA/VILI can be widely used in poverty-stricken areas in China given to the best economic evaluation results and requiring minimum medical resources, However, due to a few discrepancies in the existing research and the region limitation, the representation for nation rural residents was deficient, as well as the generalization of their conclusions. In 2015, a nationalwide cervical cancer screening progaram launched, taking VIA/VILI, careHPV and TCT as the screening methods. But the health economics of these three screening techniques in mass population screening is still unclear. Therefore, in the present study ,we simulated the process of screening diagnosis and treatment through a model, and evaluate the epidemiological and health economical effectiveness of cervical cancer screening and provide the scientific theoretical basis for the decision-making of cervical cancer prevention in rural areas of China.

## Materials and Methods

*Markov model application*

**Figure 1:**
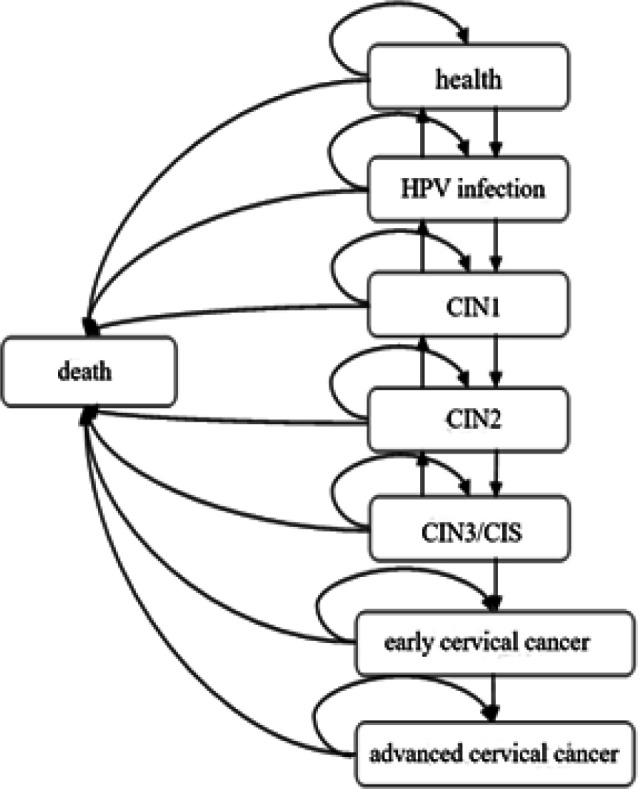
The Natural History of Carcinoma Cancer. Abbreviation: CIN, cervical intraepithelial neoplasia; CIS, Carcinoma in situ

A Markov model was in a bid to estimate the economic consequences of cervical cancer screening strategy from a societal perspective view. This model based on the natural history of cervical cancer was developed to simulate transitions between eight health states (health, HPV infection, cervical intraepithelial neoplasia [CIN] 1, CIN2, CIN3/ Carcinoma in situ [CIS], early cervical cancer, advanced cervical cancer, death) in the presence of screening (Figure 1). 

*Study population*

**Figure 2:**
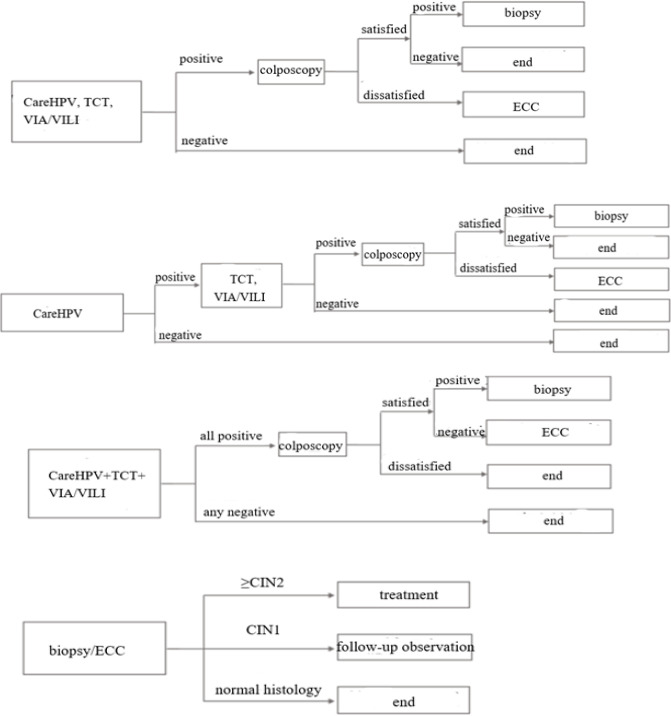
The screening strategies. Abbreviations: TCT, ThinPrep cytology test; VIA/VILL, visual inspection with acetic acid/ Lugol’s iodine; LSIL, Low-grade Squamous Intraepithelial Lesion; ECC, endocervical curettage; CIN, cervical intraepithelial neoplasia

Based on the 2015-2018 cervical cancer screening technology and demonstration research project for rural areas in China. This program selected 11 representative regions countrywide and chose a medical institution in each area as rural grass-root screening point. 3,000 women aged 35-64 were recruited from each base site through convenience sampling. Inclusion criteria are as follows: 1) Local rural household registration; 2) The history of sexual activity; 3) No history of cervical cancer and have complete cervix; 4) No symptoms of clinical pregnancy. Informed consent was obtained from all participants (The screening processes are shown in Figure 2). Random Numbers were generated by the computer to divide the subjects into careHPV, TCT and VIA/VILI respectively.

(1) Control group: women in the control group will develop cervical cancer naturally without intervention measures. The natural progression of cervical cancer goes through the following eight states: health, HPV infection, cervical intraepithelial neoplasia (CIN) 1, CIN2, CIN3, early cervical cancer, advanced cervical cancer (death from cervical cancer), and death from other diseases. Markov model of a cohort of 100,000 women was constructed according to the natural development process of cervical cancer. The model started at 35 years old and ended at 64 years old,women in the unscreened group will develop cervical cancer naturally due to no intervention measures. The natural progression of cervical cancer goes through the following eight states: health, HPV infection, cervical intraepithelial neoplasia (CIN) 1, CIN2, CIN3, early cervical cancer, advanced cervical cancer (death from cervical cancer), and death from other diseases. Markov model of a cohort of 100,000 women was constructed according to the natural development process of cervical cancer. The model started at 30 years old and ended at 79 years old with 1 year as a cycle. 

(2) Screened group: a Markov model of various screening regimens was established on the basis of the control group model. According to the comprehensive information of rural economic development and expert advice, 6 screening methods were evaluated including three separate methods (careHPV, TCT, VIA / VILI) and three combined methods (careHPV in series with VIA / VILI , careHPV in series with TCT , and threemethods in parallel; specific description in Table 1). Each method was executed with an certain interval which was defined as every 1, 3 and 5 years respectively Screening starts at 35 years and ends at 64 years. Patients with positive results were confirmed by pathological biopsy under colposcopy, and patients with CIN2 or above lesions were treated with clinical standard (screening process showed in Figure 2). 

*Screening test*

VIA/VILI screening method has been proved to be an effectively detection of cervical cancer and precancerous lesions. Especially in resource-limited settings, VIA/VILI screening, which is simple, easy to learn, and requires a minimum of infrastructure, will provide screening coverage to a larger percentage of the population than other methods. However, the sensitivity and specificity of the VIA/VILI test vary greatly in different studies large partly attribute to the diagnostic ability of the physician (Sankaranarayanan et al., 2004; Sankaranarayanan et al., 2012; Lee et al., 2016). TCT different from traditional Pap smear was a new cytological method. Liquid-based cytology improved the sample collection rate and evenly distributed the cells on the slides by controlling the cell over lapping density, which was not only conducive to reading and evaluation, but also improved the sensitivity of detecting the degree of cervical lesions (Arbyn et al., 2008; Sun et al., 2013). CareHPV was developed laying on the second generation hybridization capture technique (HC2 detection) and it can identify HPV16, 18 and other high-risk HPV types with high sensitivity and specificity (Kelly et al., 2017). This method was recommended as well given to the safety and accuracy (Qiao et al., 2008). 

*Markov model parameters*

Screening cost: Based on expert opinions and the actual situation of the screening site, a cost questionnaire was developed from the perspective of micro-cost method. The questionnaire included direct medical cost, direct non-medical cost and indirect cost. Among them, the direct medical cost was the cost of all resources (including consumables, equipment, human, etc.) used in the process of screening and diagnosis; Direct non-medical costs are mainly transportation costs; Indirect costs included project preparation, management, water and electricity supplies. 

Treatment cost: The patients with CIN2 or above were randomly selected as samples in each medical unit to inquiry the treatment costs of cervical cancer at each pathological stage in conformity to the principles of clinical diagnosis and treatment. The direct medical expenses are determined by referring to the hospital charging system. The direct non-medical expenses and indirect costs of patients were obtained by telephone interview, including nutrition, accommodation, transportation expenses, as well as the economic loss caused by the missed work for patient treatment.

Other parameters: according to some high quality literature and expert opinion, the sensitivity and specificity of different screening methods were obtained (Zhao et al., 2012; Jose et al., 2014; Liu et al., 2016). TCT was 47.1% and 96.6%, careHPV 81.3% and 88.3%, VIA/VILI 55.1% and 88.2%. The initial probability data of each pathological stage was derived from a series of cervical cancer screening studies conducted by the Cancer Institute of the Chinese Academy of Medical Sciences (CICAMS) from 1999 to 2008 (Zhao et al., 2012). Chinese health statistics yearbook and other data were searched to obtain the incidence, mortality and all-cause mortality of cervical cancer among Chinese women (National Bureau of Statistics, 2018).The transfer probability between different pathological stages was obtained from domestic and foreign literatures on the natural history of cervical cancer, and the transfer probability was fine-tuned according to the results of model operation (Ci et al., 2011).Quality of life (QOL) was collected using EuroQol five dimensions questionnaire-EQ-5D-from 194 patients with cervical cancer and precancerous lesions who visited west China second hospital of Sichuan university and Sichuan cancer hospital from 2010 to 2011. 

*Statistical analysis*

TreeAge Pro 2011 software was used to establish the screening strategy Markov model. Data derived from the model was sort out, analyzed and made charts by Excel 2013. Besides, the number of incident and death was collected by loading VBA (Visual Basic for Applications).

*1) Epidemiological evaluation*

The results of control group and screened groups were recorded by simulating a cohort of 100,000 people. The morbidity and mortality of CIN3, early cervical cancer and advanced cervical cancer were calculated. The risk of morbidity and mortality was compared between the screening group and the unscreened group to determine the epidemiological effect after the implementation of each screening program.

*1) Health economics evaluation*

Cost-effectiveness Ratios (CER) were used to estimate all the screening strategies relative to control group according to the cost-effectiveness evaluation criteria recommended by WHO (World Health Organization, 2001); Highly cost-effective (CER<China’s Gross Domestic Product, GDP per capita); Moderate cost-effective (CER>1-3 times of China’s GDP per capita); No cost-effective (CER>3 times of China’s GDP per capita). Incremental cost-effectiveness ratio (ICER) and efficiency curve were applied as well to evaluate the relative economic and effective ones among all screening strategies. Strategies lying on the efficiency curve are either less costly and more effective (strongly dominant) or more costly but more cost-effective (weakly dominant) than those lying to the left side of the curve. The slope of the efficiency curve (also the ICER) will be more gentled when the net gain in the life expectancy per Yuan is greater. By introducing QOL at each pathological stage into the model, the value of quality adjusted life years (QALY) can be obtained (QOL= QALY/actual survive time). Cost-utility analysis was conducted by calculating cost-utility ratio (CUR) with quality adjusted life years (QALY). The algorithm was similar to the CER. The benefit analysis was project benefit recommended by expert. Project benefit>0 was defined as the economic scheme. Besides, the discount rate of 3% recommended by WHO was used for analysis.

## Results

*Epidemiologic evaluation*

From 35 to 79 years old, there were 918 cases and 317 deaths of cervical cancer in the control group. The number of cervical cancer patients in the screening group ranged from 18 to 427, and the cumulative number of cervical cancer deaths ranged from 4 to 141. Each screening strategy decreased the risk of morbidity and mortality compared to the control group.The cumulative risk of incident and death of cervical cancer were reduced by more than 53% and 55%, respectively. With the increase of screening interval in the same screening program, the cumulative reduction risk of CIN3/CIS, incident and death descended gradually. The descending order of epidemiological effects was HPV-TCT-VIA/VILI, careHPV_1, HPV+VIA/VILI_1, TCT_1, HPV-TCT-VIA/VILI_3, HPV+TCT_1, VIA/VILI_1, careHPV_3, HPV-TCT-VIA/VILI_5, careHPV_5 and others. Among these, careHPV_1 similar to the parallel connection every 1 year achieved highest epidemiological effects via reducing the cumulative risk of onset and death by more than 98 % (Table 2).

*Health-economics evaluation*

**Figure 3:**
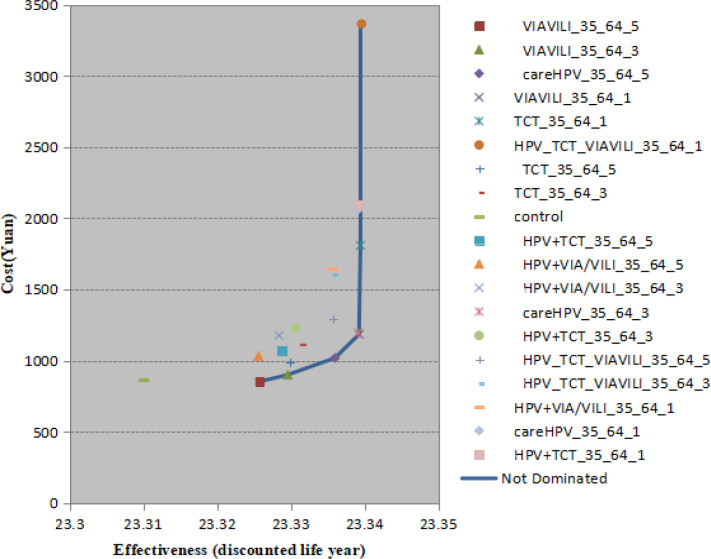
The Efficiency Curve. Strategies lying on the efficiency curve are either less costly and more effective (i.e., strongly dominant) or more costly but more cost-effective (i.e., weakly dominant) than those lying to the left side of the curve. The slope of the efficiency curve (also the ICER) will be more gentled when the net gain in the life expectancy per Yuan is greater

The total screening cost per capital of careHPV, including direct and non-direct cost, close to that of TCT, was threefold to VIA/VILI (Table 3). We calculated the CER, CUR and benefit for each strategy in relation to no intervention. CER among all the screening strategy was ranged from -756.34 to 113,040.3 Yuan per year. According to the WHO’s recommendation and Chinese GDP per capita (59,660Yuan), all the screening programs have cost effectiveness. Except careHPV in series with TCT and the three methods parallel connection every 1 year, the CERs of other screening strategies were below one time GDP per capita, it could be considered as highly cost-effective strategies. The results of CURs ranged from -169.91 to 20,163.75 Yuan per QALY are consistent with those of CERs analysis. For all strategies, the relatively decreasing order of CER and CUR was VIA/VILI_5, VIA/VILI_3, careHPV_5, TCT_5, HPV+VIA/VILI_5, TCT_3, VIA/VILI_1, careHPV_3 and others. Of notes, CUR combined curative effect with QALY was more conducive to reflect the quality of life comprehensively and accurately. However, in practice, only considering the CER cannot fully demonstrate the benefits and drawbacks of each screening strategy. The results of ICER and efficiency curve provided a direct and further explanation about whether the screening method is optimal or not. According to the model outcome, domination screening strategies were identified including HPV-VIA/VILI-TCT_1, TCT_1, careHPV_5, VIA/VILI_1, VIA/VILI_3, VIA/VILI_5. The higher slope of the line (equivalent to higher ICER) of the first two program means that compared with the previous cost-neighboring one, the extra cost of this project was very high (>1000,000 Yuan/life year) to saves one life year. In addition, careHPV_3 and VIA/VILI_1 almost coincided in the curve (Figure 3). 

In terms of the benefits analysis, the results ranged from -1,629 to 996 Yuan. Except careHPV_1, HPV+TCT_1, HPV+ VIA/VILI _1, HPV-TCT-VIA/VILI_1, all rest strategies has cost-benefit (benefit>0) (Table 4).

**Table 1:** The Description of Screening Strategy

N	screening method (abbreviation)	starting age	end age	interval time	screening method
1	TCT_1	35	64	1	TCT
2	careHPV_1	35	64	1	careHPV
3	VIA/VILI_1	35	64	1	VIA/VILI
4	careHPV+TCT_1	35	64	1	careHPV in series with TCT
5	careHPV+VIA/VILI_1	35	64	1	careHPV in series with VIA/VILI
6	HPV-TCT-VIA/VILI_1	35	64	1	careHPV, VIA and TCT in parallel
7	TCT_3	35	64	3	TCT
8	careHPV_3	35	64	3	careHPV
9	VIA/VILI_3	35	64	3	VIA/VILI
10	careHPV+TCT_3	35	64	3	careHPV in series with TCT
11	careHPV+VIA/VILI_3	35	64	3	careHPV in series with VIA/VILI
12	HPV-TCT-VIA/VILI_3	35	64	3	careHPV, VIA and TCT in parallel
13	TCT_5	35	64	5	TCT
14	careHPV_5	35	64	5	careHPV
15	VIA/VILI_5	35	64	5	VIA/VILI
16	careHPV+TCT_5	35	64	5	careHPV in series with TCT
17	careHPV+VIA/VILI_5	35	64	5	careHPV in series with VIA/VILI
18	HPV-TCT-VIA/VILI_5	35	64	5	careHPV, VIA and TCT in parallel

Abbreviation: TCT, ThinPrep cytology test; VIA/VILL, visual inspection with acetic acid/ Lugollo iodine

**Table 2:** Results of Epidemiological Evaluation of Screening Program

Screening strategy	The number of CIN3/CIS	The number of cases	The number of death	Reduction in cumulative risk of CIN3/CIS (%)	Reduction in cumulative risk of cases (%)	Reduction in cumulative risk of death (%)
Control	7,081	918	317	-	-	-
TCT_1	2,227	49	5	68.55	94.66	98.42
HPV+TCT_1	2,612	62	8	63.11	93.25	97.48
careHPV_1	1,253	18	4	82.3	98.04	98.74
HPV+VIA/VILI_1	2,316	46	5	67.29	94.99	98.42
VIA/VILI_1	3,872	68	10	45.32	92.59	96.85
HPV-TCT-VIA/VILI_1	984	13	4	86.1	98.58	98.74
TCT_3	4,056	222	70	42.72	75.82	77.92
HPV+TCT_3	4,442	281	78	37.27	69.39	75.39
careHPV_3	2,928	84	11	58.65	90.85	96.53
HPV+VIA/VILI_3	4,152	237	68	41.36	74.18	78.55
VIA/VILI_3	3,751	177	45	47.03	80.72	85.8
HPV-TCT-VIA/VILI_3	2,564	50	10	63.79	94.55	96.85
TCT_5	4,871	357	127	31.21	61.11	59.94
HPV+TCT_5	5,199	427	141	26.58	53.49	55.52
careHPV_5	3,837	165	45	45.81	82.03	85.8
HPV+VIA/VILI_5	4,956	376	126	30.01	59.04	60.25
VIA/VILI_5	4,601	299	94	35.02	67.43	70.35
HPV-TCT-VIA/VILI_5	3,482	118	29	50.83	87.15	90.85

Abbreviation: TCT, ThinPrep cytology test; VIA/VILL, visual inspection with acetic acid/ Lugollo iodine; CIS, carcinoma in situ; CIN, cervical intraepithelial neoplasia; “_1”: screening every 1 year; “_3”: screening every 3 years; “_5”: screening every 5 years. “+”:in series with; “¬-”: in parallel

**Table 3:** Screening and Diagnosis of Cervical Cancer and Its Precancerous Lesions in Rural Areas (Yuan/Person)

Screening method	direct medical cost			Indirect medical cost	Indirect cost	
	consumables	equipment	human			total
VIA/VILL	3.38	0.09	8.66	3	3.03	18.16
TCT	34.22	1.11	11.60	3	11.73	61.67
CareHPV	42.82	0.33	10.17	3	13.33	69.65
CareHPV+ VIA/VILI	46.20	0.42	18.83	6	16.36	87.80
CareHPV+ TCT	74.60	1.34	15.87	3	22.95	117.77
Colposcopy	3.57	1.60	14.85	3	5.01	28.04
Biospy	9.95	4.83	17.48	0	8.06	40.32

Abbreviation: TCT, ThinPrep cytology test; VIA/VILL, Visual inspection with acetic acid/ Lugol’s iodine; “+”, in series with;

**Table 4:** Results of Health Economic Evaluation of Screening Program

Screening strategy	Discounted cost (Yuan/person	Discounted QALY (year/people)	CER (Yuan/year)	ICER (Yuan/ year)	CUR (Yuan/ year)	Benefits (Yuan)
Control	951.33	23.17	-	D	-	-
TCT_1	2,024.77	23.29	44,148.64	3095561.4	8,487.8	136
HPV+TCT_1	2,382.34	23.29	60,794.51	D	11,968.27	-260
careHPV_1	2,341.46	23.31	54,443.33	D	9,864.28	-120
HPV+VIA/VILI_1	2,228.69	23.29	52,905.08	D	10,229.67	-77
VIA/VILI_1	1,323.89	23.30	15,037.92	51112.48	2,840.27	859
HPV-TCT-VIA/VILI_1	3,859.72	23.31	113,040.31	10739073.85	20,163.75	-1,629
TCT_3	1,241.23	23.26	14,821.02	D	3,197.48	683
HPV+TCT_3	1,377.7	23.25	23,996.55	D	5,311.5	457
careHPV_3	1,336.45	23.28	16,264.22	D	3,270.17	792
HPV+VIA/VILI_3	1,314.63	23.25	18,990.18	D	4,122.35	588
VIA/VILI_3	9,93.26	23.27	2,009.43	12535.88	425.16	996
HPV-TCT-VIA/VILI_3	1,861.71	23.29	37,018.75	D	7,274.01	312
TCT_5	1,098.29	23.24	9,060.94	D	2,078.59	660
HPV+TCT_5	1,191.48	23.23	16,765.99	D	3,961.52	472
careHPV_5	1,131.13	23.27	8,350.04	17769.62	1,783.8	891
HPV+VIA/VILI_5	1,146.07	23.23	12,365.95	D	2,856.47	588
VIA/VILI_5	937.92	23.25	-756.34	*	-169.91	895
HPV-TCT-VIA/VILI_5	1,448.31	23.34	21,586.26	D	50.83	648

Abbreviation: TCT, ThinPrep cytology test; VIA/VILL, visual inspection with acetic acid/ Lugol’s iodine; QALY, quality-adjusted life year ICER: incremental cost-effectiveness ratio; CER, cost-effectiveness ratio; CUR, cost-utility ratio; D, dominated strategy. “*”, The scheme has the lowest cost and no incremental cost; “_1”, screening every 1 year; “_3”, screening every 3 years; “_5”, screening every 5 years; “+”, in series with; “¬_”, in parallel

## Discussion

To the best of knowledge, the present study was the first to nationally and comprehensively estimate different screening strategies of cervical cancer in rural China. Giving an overall consideration to the information on the risks of HPV infection, screening and treatment procedures and the costs associated with screening, diagnosis and treatment, careHPV 3 or 5 years and VIA/VILI_1 or 3 years eventually considered as dominant strategies in rural China.

Our results showed the shorter the interval, the better the epidemiological results, but also the higher the cost. Therefore, the optimal screening program suitable for Chinese rural areas should be judged comprehensively based on the epidemiological and health economics effects. From ICER analysis, we found HPV-TCT-VIA/VILI_1, TCT_1, VIA/VILI_1, careHPV_5, VIA/VILI_3, VIA/VILI_5 were dominant strategies. Among these, HPV-TCT-VIA/VILI_1, and TCT_1 gained remarkable epidemiologic effects by making a reduction of cumulative risk of incident and death of cervical cancer more than 94%. However, both were not recommended to implement in economic lagging regions due to the highly ICER (>1000,000 Yuan/life year) and poor project benefit. 

VIA/VILI_5 was also removed for moderate epidemiological effects by making a reduction of cumulative risk of incident and death of cervical cancer less than 70%, which may account for the lower Youden index (0.43) compared with other strategies. While VIA/VILI 1 or 3 years has shown its superiority in these analyses. Although the screening frequency increased, VIA/VILI still provided highly cost-effectiveness probably driven by the lowest screening cost (only a third of the cost of careHPV and TCT). Previous Chinese study conducted in poverty-stricken region among women aged 35-65 found VIA/VILI performed the best in terms of health economic evaluation results, as the cost of per positive case detected was 8,467.9 RMB, which was 24,503.0 RMB lower than that for TCT and 5,755.9 RMB lower than that for the HPV test. In addition, it also obtained substantial epidemiological effect. VIA/VILI averted the greatest number of YLD every year and both the positive detection rate and the positive predictive value were higher in the VIA/VILI group than HPV test and TCT (Shi et al., 2011). That was fairly consistent with our study. They suggested lower sensitivity could be compensated by higher screening rate. Besides, VIA/VILI, which is simple, easy to learn, and requires a minimum of infrastructure, will be an effective way to increase cervical cancer screening coverage (Lee et al., 2016). These advantages can provide a framework to integrate VIA/VILI_1 screening into primary healthcare services in rural areas that lack medical resources and burden a high incidence of cervical cancer accord with the WHO’s advice (World Health Organization, 2001). In addition, our findings suggested careHPV_3 and VIA/VILI_1 almost coincided on the dominance curve. And the former one was also considered as highly cost-effective project when it comes to CER. In the epidemiology term, careHPV_3 similar to VIA/VILI_1 made comparably descend in the cumulative risk of onset and death of cervical cancer by more than 90% and 96% respectively. Fewer screenings could have the same effect which may large partly attribute to more opportunities for early detection and treatment. A recently comprehensive review reported the careHPV has shown good sensitivity and specificity for the detection of CIN2+ (88.1% and 83.7%, respectively) and CIN3+ (90.3% and 85.3%, respectively) (Kelly et al., 2017). The benefits of careHPV were more pronounced at the same time interval. Another study conducted in ShanXi province reported careHPV was more effective to reduce cancer incidence and mortality by 48% and 54% respectively than VIA (37%, 43%) from the age 35 years (Shi et al., 2011). Of note, simplification of careHPV procedure compared with the conventional HPV test has resulted in a faster running time, a wider scope for the Operator and hospital conditions (Qiao et al., 2008). A Thailand study confirmed the superiority of careHPV in low-resource settings given to the great feasibility, flexibility, and acceptability as well (Trope et al., 2013). Thus, careHPV_3 was also recommended as a dominant screening strategy. The remaining two dominant schemes, careHPV_5 and VIA/VILI_3 gained analogous effect regardless of epidemiology or economy. Epidemic effect is lower than VIA/VILI_1 and careHPV_3, but more economical. Hence, from a full-fledged point of view, VIA/VILI_1 and careHPV_3 was priority to be a preferred and rapid screening methods in rural with relatively adequate resources. VIA/VILI_3 and careHPV_5 turn to be the dominate protocols in resource-contained areas in China rural.

Some limitations in the present study should be acknowledged. Screening costs and treatment costs were collected on the basis of the 2015 national health specifically. Data collected from each basic level point data were somewhat difference. It may large partly attribute to the economic level discrepancy in the north and south China rural. Although we adjusted by the comparison of local GDP and national data. There are still some inevitable deviations. In addition, it is difficult to avoid information bias considering economically sensitive issues in the investigation. Furthermore, in this study, the establishment of screening program Markov model was a relatively static process. Dynamic models should be established to adjust parameters for analysis. Despite the shortcomings of survey data, they represent the best available data using similar measures across different regions. It still can provide a reference and lay the groundwork for future budget planning and cost-effectiveness evaluations of planned potential large-scale cervical screening programs in China rural.

In conclusion, all screening strategies in this study have cost-effectiveness and significantly reduce the health and economic burden of cervical cancer. Women aged 35 to 64 should be encouraged to actively participate in screening. In order to make more reasonable use of limited medical resources, VIA/VILI every 1 or careHPV every 3 years were regard as practical and effective primary screening methods for public-health cervical cancer prevention programs, especially in well-resourced settings in rural. While VIA/VILI every 3 years and careHPV every 5 should implemented in China rural and may be affordable and cost-effective options for public-health programs in poverty-stricken areas in rural. 
