# Supplementary material for: Epidemiologic and Health Economic Evaluation of Cervical Cancer Screening in Rural China
Source: Asian Pac J Cancer Prev. 2020 May;21(5):1317–25. doi: 10.31557/APJCP.2020.21.5.1317 (PMC7541874; doi:10.31557/APJCP.2020.21.5.1317)
Supplement: Supplement [file APJCP-21-1317-s001.pdf]

Supplementary Table 1 initial probability of each process of cervical cancer and its precancerous lesions

health status	health	HPV infection	CIN1	CIN2	CIN3	early cervical cancer	advanced cervical cancer	death
initial probability	0.847	0.0983	0.0285	0.0139	0.0111	0.0006	0.0001	0

Abbreviation: CIN: cervical intraepithelial neoplasia;

Supplementary Table 2 death rates for different disease states in different age groups

age（year）	health-CIN3/CIS	early cervical cancer	advanced cervical cancer
35-39	0.000696	0.000713	0.177808
40-44	0.001074	0.001113	0.177808
45-49	0.001627	0.001683	0.177808
50-54	0.002745	0.002807	0.177808
55-59	0.004226	0.004281	0.177808
60-64	0.007399	0.007464	0.177808
65-69	0.012906	0.01297	0.177808
70-74	0.023987	0.024062	0.177808
75-79	0.039966	0.040063	0.177808

Abbreviation: CIN: cervical intraepithelial neoplasia; CIS: carcinoma in situ;

Supplementary Table 3 sensitivity and specificity of screening methods in rural areas

index	TCT	careHPV	VIA/VILI
sensitivity	0.471	0.813	0.551
specificity	0.966	0.883	0.882

Abbreviation: TCT: ThinPrep cytology test; VIA/VILL: Visual inspection with acetic acid/ Lugol’s iodine;

Supplementary Table 4 transition probabilities between status of Markov model

initial status	transfered status	1 year transition probability
health	health	0.910-0.990
	HPV infection	0.010-0.090
HPV infection	health	0.430-0.490
	HPV infection	0.435-0.460
	CIN1	0.060-0.120
CIN1	HPV infection/health	0.253
	CIN1	0.602
	CIN2	0.145
CIN2	CIN1	0.226
	CIN2	0.666
	CIN3/CIS	0.108
CIN3/CIS	CIN2	0.030-0.080
	CIN3/CIS	0.895-0.935
	early cervical cancer	0.015-0.035
early cervical cancer	early cervical cancer	0.82
	advanced cervical cancer	0.18

Abbreviation: CIN: cervical intraepithelial neoplasia; CIS: carcinoma in situ;

Supplementary Table 6 the total cost of treatment in different pathological stages in rural areas

pathological stages	direct medical cost (yuan/ person)	Indirect medical cost (yuan/ person)	Indirect cost (yuan/ person)	Total (yuan/ person)
**CIN2**	3,894.56	1,033.3	3,521.25	**8,449.1**
**CIN3**	5,893.33	1,495.31	6,481.6	**13,870.24**
early cervical cancer	23,372.94	5,901.69	16,249.11	**45,523.75**
advanced cervical cancer	35,630.48	11,894.34	37,934.18	**85,459**

Abbreviation: CIN: cervical intraepithelial neoplasia; CIS: carcinoma in situ;
